# Hook Plate Versus Distal Locking Plate for the Fixation of Unstable Distal Clavicle Injuries, Outcomes and Complications: A Meta-Analysis

**DOI:** 10.7759/cureus.30806

**Published:** 2022-10-28

**Authors:** Mohamed Elrih, John Quinlan

**Affiliations:** 1 Trauma and Orthopaedics, Tallaght University Hospital, Dublin, IRL

**Keywords:** distal clavicle injuries, distal locking plate, acromioclavicular dislocation, distal clavicle fracture, hook plate

## Abstract

Numerous interventions are advised for the surgical management of distal clavicle fractures. Hook plate (HP) and distal locking plate (DLP) are among the commonly used techniques; nonetheless, no single procedure is deemed to be the benchmark treatment. Thus, the aim of the study is to conduct a comparative analysis, hopefully, to recommend the superior method between the two operations.

PubMed, Embase via Ovid and Web of science were electronically searched between January 2000 and to date for studies directly comparing HP to DLP. Comparative retrospective/prospective and randomized studies were incorporated. Constant-Murley score “CMS” at a minimum of 12 months, pain visual analogue scale “VAS”, coracoclavicular distance “CCD” and reported complications were analysed. Review manager software was used for the statistical analyses.

The total number of patients was 523; 274 (52.3%) with HP and 249 (47.6%) with DLP, 81 of which were associated with CC reconstruction. The mean follow up was 38.7 and 37.03 months for HP and DLP, respectively. CMS leaned towards the DLP group with no statistically significant difference (P=0.06). VAS was in favour of the DLP with again no statistically significant difference (P=0.12). In terms of CCD, the comparison favoured the HP with a lesser CCD postoperatively and a statistically significant difference (P<0.05). Complications were significantly higher in the HP group (P<0.0001).

Contrary to our hypothesis, though HP did show a better radiological outcome; nonetheless, DLP did demonstrate a better functional result with a lesser rate of complications and the ability to retain the implant avoiding a second surgery.

## Introduction and background

Distal clavicle fractures account for about 10%-30% of all clavicle fractures [1,2] and 50% of nonunion cases [3]. Injuries to the lateral third of the clavicle could lead to disruption of the coracoclavicular (CC) ligament compromising the stability of the fracture and the acromioclavicular joint (ACJ), thus, resulting in higher rates of malunion and nonunion [1].

A hook plate (HP) is commonly used as a spanning device in the management of distal clavicle fractures, it has the capability of restoring the CC height and maintaining stability by being hooked under the acromion [1,4]. Nonetheless, it comes with its own share of complications, such as shoulder impingement causing long-term pain and decreased abduction, erosion of the acromion leading to osteolysis, arthritis of the ACJ, and the need of a second operative visit for the removal of the metal [4,5]. On the other hand, isolated distal locking plates (DLPs), though not technically associated with the aforementioned complications, it had been reported to have higher rates of delayed union and nonunion of up to 10% along with the incapacity of fully restoring the CC height [4,6,7].

Several kinds of operative interventions in addition to HP and DLP are available for the management of distal clavicle injuries, however, no single procedure is deemed to be the benchmark treatment [8]. Studies comparing HP to DLP are relatively limited, thus, we aimed to conduct a comprehensive comparative evaluation of the outcomes and reported complications of both techniques, hopefully, to be able to recommend a superior method from the two procedures.

## Review

Materials and methods 

The study had been organized as per the recommendations of the Cochrane Handbook for Systematic Reviews of Intervention and in accordance with the Preferred Reporting Items for Systematic Reviews and Meta-analyses (PRISMA), network meta-analysis extension [9,10].

*Search Strategy and Selection of Studies* 

An electronic databases search in PubMed, Embase via Ovid and Web of science between January 2000 and to date was performed to identify studies directly comparing HP to DLP. No restriction on the number of cases included in the reviews with the elimination of cadaveric and duplicated studies. The search was limited to the English language. Keywords for the search were “clavicle hook plate”, “distal clavicle fracture”, “distal clavicle locking plate” and “acromioclavicular dislocation”, in combination with the Boolean operators “AND” or “OR”. The main author independently examined the resulting titles and abstracts to extract relevant articles.

Eligibility Criteria and Study Selection

All titles were initially screened for comparative studies in the management of distal clavicle fractures and/or ACJ dislocation. Selected abstracts were subsequently examined for detailed evaluation and confirmation of inclusion criteria.

Studies were included in the meta-analysis if they fulfilled the following criteria: skeletally mature patients with acute (less than 8 weeks) unstable (Neer type-II, III and V) [10] distal clavicle fracture, intervention and comparator between HP and DLP with or without coracoclavicular (CC) reconstruction, comparing postoperative outcomes like Constant-Murley score (CMS) at a minimum of 12 months, pain visual analogue scale (VAS) at one year follow up, coracoclavicular distance (CCD) and reported complications. Comparative retrospective/prospective and randomized studies were incorporated from the literature search with appropriate data to extract and pool for the statistical analysis {total number of subjects in each intervention, reported mean and standard deviation (SD) for the continuous outcomes, and number of cases in each event (complication) for the dichotomous outcomes} The timing of removal of the HP was not clearly documented in the included studies.

Noncomparative articles and studies not including the outcome measures of interest were excluded from the meta-analysis. Abstracts and studies without fully available text were also excluded. Confusion about the literature was consulted with the supervisor for expert opinion and clarification.

Data Analysis

Review manager software, 5.4.1 edition, was used for the statistical analyses. A mean difference (MD) with a 95% confidence interval (CI) was used for the continuous data. Moreover, a risk ratio (RR) with 95% CI was used for the dichotomous outcomes. P-value and I2 measurements utilizing the chi-squared test were used to assess the statistical heterogeneity. Random-effects standard is used with statistical results of P<0.05 or I2 > 50%. Pooling of continuous outcomes was established if there were at least four studies comparing that result.

Results

Data Extract

The main author extracted the available targeted data from the included articles. Data were initially saved as tables in a pre-arranged and designed Microsoft Word document for later easy collection and entry into statistical software and a Microsoft Excel worksheet for chart formation. The data extracted included the first author, year of publication, characteristics of patients (age, sex) and a number of patients in each group (HP, DLP). SD and reported mean of the continuous outcomes (CMS, VAS and CCD) of each group were extracted. In the continuous data, if the mean was only reported then the SD was calculated as a range of (maximum - minimum)/4. Dichotomous outcomes represented by the reported complications {subacromial osteolysis, shoulder pain, delayed union, nonunion, malunion, impingement, heterotopic ossification (HO), implant loosening, ACJ subluxation, osteoarthritis (OA) changes, loss of reduction and periprosthetic fractures} were all extracted.

Characteristics of Included Data

**Figure 1:**
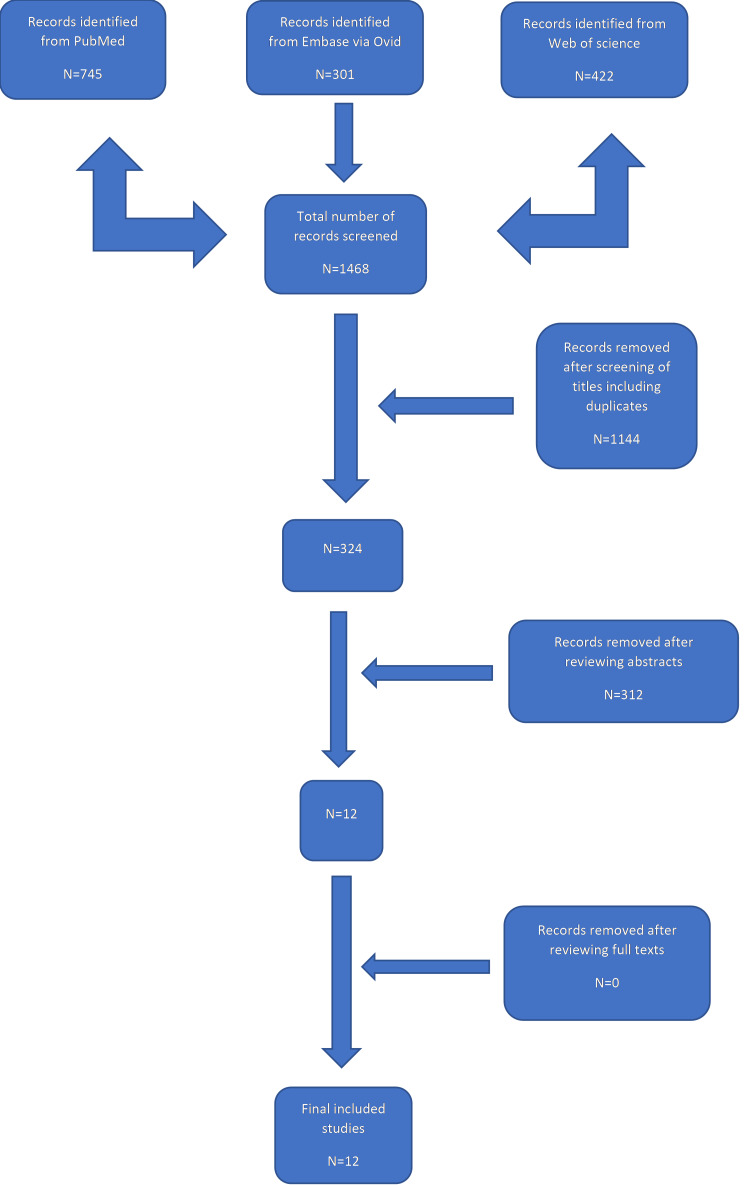
Flow diagram of the studies selection process

Database search yielded a total of 1,468 records (Figure 1): 745 records from PubMed database, 301 from Embase via Ovid and 422 from Web of Science. 1,144 studies were removed after reviewing the titles which showed irrelevant studies (not addressing the topic) and duplicated studies. Three hundred twelve were removed after reviewing abstracts. Twelve texts were fully screened and considered eligible for the inclusion in this comparative meta-analysis [4,8,11-20].

One of the main reasons for excluding studies after reviewing the abstracts was the comparison between HP and other forms of fixation, for example: tension band wire or isolated CC reconstruction. The total number of patients included in the study was 523 (Table 1): with 274 (52.3%) patients having HP and 249 (47.6%) having DLP (81 of which with CC reconstruction). The mean follow-up was 38.7 months for the HP group, and 37.03 months for the DLP group. The overall mean for the age was 42.5 years (ranging from 34.5 to 56.3years) for the HP group, and 38.9 years (ranging from 32 to 55.2 years) for the DLP group. Male to female percentage was 66.9% (350/523) to 33.07% (173/523).

**Table 1:** General characteristics of the included studies

Study	Male	Female	Age (HP : DLP)	HP	DLP	Type of Study
Wang et al. [11]	40	24	37.03 ± 10.01 : 38.77 ± 8.55	33	31	Retrospective
Zhang et al. [8]	37	29	41.1 ± 10.3 : 42.5 ± 10.7	30	36	Retrospective
Li et al. [12]	27	17	51.7±17.1 : 55.2±16.3	26	18	Retrospective
Erdle et al. [14]	28	4	44.3+_14.9 : 43.7_+13.7	19	13	Retrospective
Seo et al. [4]	53	29	43+_15.8 : 45.6+_18.2	54	28	Retrospective
Yoon et al. [15]	45	15	39.9 ± 14.8 : 47.4 ±14.8	28	32	Retrospective
Gutman et al. [16]	9	3	56.3 ± 7.2 : 47.0 ± 16.7	12	8	Retrospective
Das et al. [17]	22	10	44.35: 38.93	17	15	Prospective
Hickland et al. [18]	14	12	41.5 (36.8–53.3) : 41.0 (30.8–53.8)	10	16	Retrospective
Bandebuche et al. [19]	42	18	41.4 years (range: 19-68 years)	30	30	Retrospective
Bhatia et al. [20]	13	2	34.5 years (20-57)	10	5	Retrospective
Orlandi et al. [13]	20	10	35+_11 : 32+_10	13	17	Randomized Controlled Trial
Total	350	173		274	249	

Assessment of Study Quality

Twelve comparative studies [4,8,11-20] were included, 10 retrospective, one prospective and one prospective randomized comparative study. All were reviewed and pooled to conduct this meta-analysis. The Methodological Index for Non-Randomized Studies (MINORS) was used to assess the risk of bias in nonrandomized studies [21]. The index included the following items to evaluate and note each score (Table 2): clearly stated aim, Inclusion of consecutive patients, Prospective collection of data, Endpoints for the aim of the study, Unbiased assessment of study endpoint, Appropriate follow-up period, Follow-up loss <50%, Prospective calculation of study size, Adequate control group, Contemporary groups, Equivalent baseline of groups and Adequate statistical analyses. As per the MINORS, the items are scored 0 (if not reported), 1 (if reported but inadequate) or 2 (if reported and adequate). The overall ideal score is 24 for comparative studies [21]. The quality of the comparative study is considered poor if scoring 0-12, fair quality if scoring 13-18 and excellent quality if scoring 19-24 [22]. For the randomized study, the risk of bias was evaluated as per the PRISMA guidelines (Table 3) by assessing: Adequate sequence generation, Adequate allocation concealment, blinding (of participant and assessors), Addressing incomplete outcome data, Reporting of selective outcome and Assessment of extra sources of bias [9,10].

**Table 2:** Risk of bias assessment according to the MINORS tool

	Wang et al. [11]	Zhang et al. [8]	Li et al. [12]	Erdle et al. [14]	Seo et al. [4]	Yoon et al. [15]	Gutman et al. [16]	Das et al. [17]	Hickland et al. [18]	Bandebuche et al. [19]	Bhatia et al. [20]
Clear stated aim	2	2	2	2	2	2	2	2	2	2	2
Inclusion of consecutive patients	2	2	2	2	2	2	2	2	2	2	2
Prospective collection of data	0	0	0	0	0	0	0	0	0	0	0
Endpoints for the aim of study	2	2	2	2	2	2	2	2	2	2	2
Unbiased assessment of study endpoint	2	2	2	2	2	2	2	2	2	2	2
Appropriate follow-up period	2	2	2	2	2	2	2	0	1	2	2
Follow-up loss <50%	2	2	2	2	2	2	2	2	2	2	2
Prospective calculation of study size	0	0	0	0	0	0	0	0	0	0	0
Adequate control group	2	2	2	2	2	2	0	2	2	2	0
Contemporary groups	2	2	2	2	2	2	2	2	2	2	2
Equivalent baseline of groups	2	2	2	2	2	2	2	2	2	2	2
Adequate statistical analyses	2	2	2	2	2	2	2	2	2	2	2
Total score	20	20	20	20	20	20	18	18	19	20	18

**Table 3:** Risk of bias assessment for the randomized trial as per the PRISMA guidelines

	Adequacy of sequence generation	Allocation concealment	Blinding	Addressing incomplete outcome data	Reporting of selective outcome	Assessment of extra sources of bias
Orlandi TV et al. [13]	Yes	Yes	Yes	Yes	Yes	Yes

Constant Score

The constant-Murley score is considered one of the most commonly used scoring systems in the assessment of the functional outcome of the shoulder [23]. It consists of four parts; a 15-point representing pain reported by the patient, a 20-point for Activities of Daily Living, 40 points for the Range of Motion examined by the physician and a 25-point for the strength of the shoulder which is also assessed by the examiner [23]. Grading of the outcomes ranges from (<56 points) poor, (56-70) fair, (71-85) good and (86-100) very good [24].

**Figure 2:**
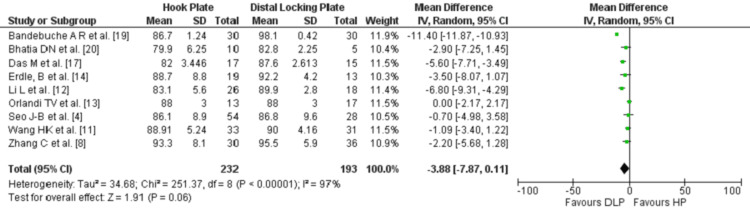
Forest plot for the comparison of Constant-Murley scores at the last follow-up between HP and DLP

Nine studies [4,8,11-14,17,19,20] out of the 12 studies included in the meta-analysis addressed the Constant score with a total of 425 patients: 232 (54.5%) and 193 (45.4%) in each of the HP and DLP groups, respectively. The study by Bhatia and Page [20] shows a “good” CMS mean in both the HP and DLP groups. Two studies, Li et al. [12] and Das et al. [17] also showed a “Good” CMS mean in the HP group, whereas the same studies showed a “Very good” score in the DLP group. The remaining six studies [4,8,11,13,14,19] demonstrated a “Very good” CMS mean in both groups (HP and DLP) with a mean score of >86. In general, the overall data collection and analyses of the CMS leaned towards the DLP group, nonetheless, there was no statistically significant difference (P=0.06), (Figure 2).

Visual Analogue Scale (VAS)

**Figure 3:**
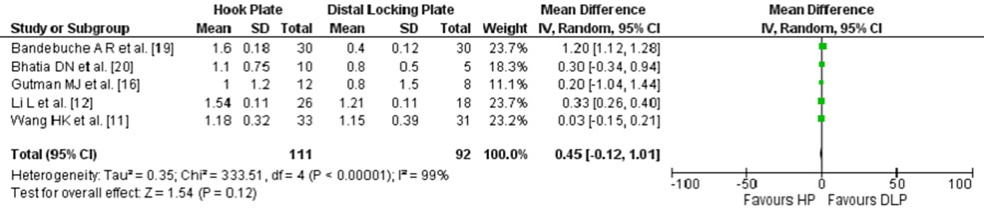
Forest plot for the comparison of the VAS at the last follow up between HP and DLP

The VAS is a psychometric measuring method used to document the level of associated symptoms of a certain disease or injury [25]. It is a rapid tool that assesses the severity of the complaints and facilitates the categorization of the outcomes [25]. Grades are made by marking on a 10cm line that symbolizes a scale ranging between “No Pain” to “Worst Pain” [26]. The scores of the VAS could be created in reflection of the final results, a VAS >5 represents more severe pain or uncontrollable symptoms, whereas <2 indicates a well-controlled symptoms [25]. Five studies [11,12,16,19,20] reviewed the VAS comparing the mean in the two groups with a mean follow up duration of 22.02 months (ranging from six to 60 months). Number of patients involved in the evaluation were 111 and 92 in the HP and DLP groups, respectively. Both groups in the included studies showed a mean score of <2, with relatively a better outcome in the DLP group favouring this type of fixation. The comparison between the two groups was again not statistically significant (P=0.12), (Figure 3).

Coracoclavicular Distance (CCD)

**Figure 4:**
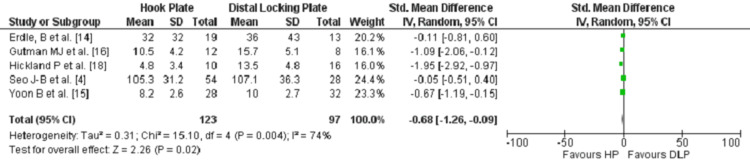
Forest plot for the comparison of the CCD postoperatively at the last follow-up between HP and DLP

Five studies [4,14-16,18] examined the mean difference of the CCD in a follow up period ranging from 0 to 5 months postoperatively, with a total of 220 patients, 123 (55.9%) in the HP group and 97 (44.09%) in the DLP group. The comparison favoured the HP with a lesser CCD postoperatively when compared to the DLP. Results were statistically significant (P<0.05), (Figure 4).

Complications

**Figure 5:**
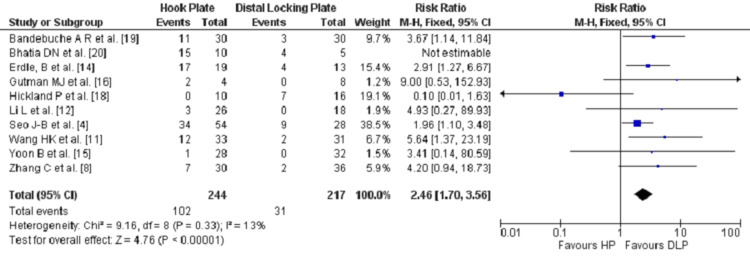
Forest plot for the comparison of the reported complications at the final review

**Figure 6:**
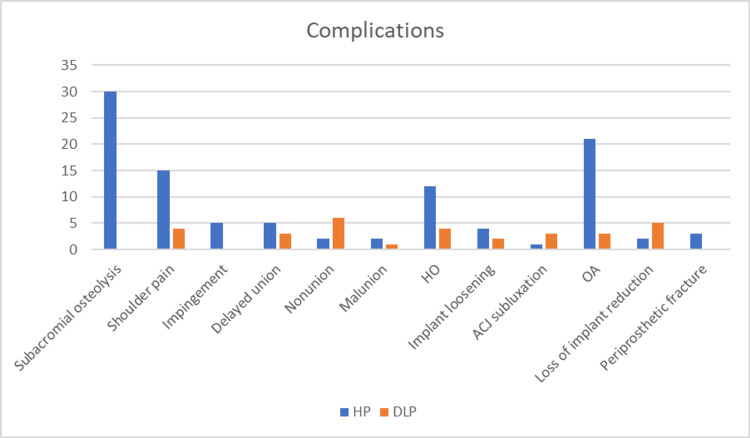
Chart comparing the numbers of complications between the two groups (HP vs DLP)

Complications were stated in ten studies [4,8,11,12,14,15,16,18-,20] out of the 12 studies included. Overall, 133 events were reported, with 102 (76.6%) in the HP group, and 31 (23.3%) in the DLP group. Clearly, the HP group showed a significantly higher rate of complications compared to the DLP group. The highest three complications reported in the HP group were Subacromial osteolysis, osteoarthritis and shoulder pain, with a count of 30, 21 and 15, respectively. In the DLP group, the highest two complications reported were nonunion and loss of implant reduction, six and five events, respectively. Hypertrophic ossification (HO) and shoulder pain were next in order in the DLP group, with four events in each. Nonunion, ACJ subluxation and loss of implant reduction were the notable complications that had higher rates in the DLP in comparison to the HP, 6:2, 3:1 and 5:2, respectively. Remarkably, the DLP group showed zero case in terms of subacromial osteolysis, impingement and periprosthetic fracture. Outcomes were statistically significant (P<0.0001) when comparing the two groups (Figure 5) and numbers were in favor of the DLP group (Figure 6).

Discussion 

The HP is considered a dynamic internal fixator, having the tip of the plate implanted deep beneath the acromion and posterior to the ACJ, with the medial part of the plate firmly fixed into the clavicle [12]. The clavicular HP acts as a lever by lifting the acromion and buttressing down the clavicle to maintain a stable fracture distally, with no interference to the rotational movement of the clavicle itself [27]. HP has an uneven mechanical distribution with higher stress forces at the ACJ, thus, leading to an increased risk of subacromial osteolysis and fracture [12,28]. The ideal depth of the hook under the acromion and the correct bending of the plate could be accomplished intraoperatively to avoid the aforementioned complications [12]. Patients with immature bone growth should be assessed carefully prior to selecting the implant of choice, as HP may affect normal bony growth and cause permanent developmental damage to the acromion [12].

The hypothesis was, that HPs theoretically reconstruct CC stability more efficiently and therefore result in superior outcomes [14]. The evaluation which was conducted in the present meta-analysis in regard to CC height did favour the HP in restoring the CCD. Nonetheless, the study also showed a number of outcomes that favoured the DLPs in comparison to the HP.

In 2018, Yoon et al. [15] conducted a study comparing the radiological outcomes for patient's post-fixation with HP and DLP (without CC reconstruction) for distal clavicle fractures. The mean CCD was noted to be significantly reduced when comparing the pre-surgical and post-surgical findings. In the HP group, it was documented to be 19.4 ± 4.3 mm preoperatively and 6.8 ± 3.3 mm postoperatively. In the DLP group, it was 17.7 ± 6.7 mm initially at presentation and 10.8 ± 2.5 mm post-surgery [15]. The decrease in the CCD was found to be statistically significant (P<0.01), with a quite margin between the two techniques favouring the HP fixation. Therefore, Yoon et al. [15] suggested that the preservation of the CC distance cannot be achieved via fracture fixation and reduction only, a stabilization of the CC ligament is considered essential to maintaining the reduction.

Hickland et al. [18] conducted a retrospective observational study comparing four techniques for the management of distal clavicle fractures. Three of these fixations are in the interest of this study including HP, isolated DLP and DLP with CC ligament reconstruction. A total of 44 patients were examined, 10 patients had HP, 16 patients had isolated DLP, and six patients had DLP with CC reconstruction. Assessment of the radiological data showed significant improvement in the postoperative CCD in the HP group when compared to the isolated DLP and DLP with CC reconstruction groups, (P<0.001) and (P=.047), respectively [18]. The study informed that isolated DLP seems to be the least efficient technique [18]. Though not significant (P=0.114), isolated DLP was found to have more incidents of ACJ malreduction when compared to other means of fixation [18]. Hickland et al. [18] advised the need for concomitant CC reconstruction in all cases associated with rupture of the CC ligament, especially, if the AC ligament is found to be unstable, as patients with CC ligament injury rely on their AC ligament for the stability of the distal fragment. Therefore, there is a high risk of ACJ dislocation/subluxation if the fracture is fixed with a DLP only [18]. All patients (100%) in the HP group underwent removal of the implant at an average operative time of 4.3 to 6.5 months. The clinical outcomes, as per Hickland et al. [18], showed better functional scores and patients’ satisfaction when the HP was removed within this period of time. On the contrary, DLP was only removed at the request of patients due to symptomatic discomfort in three (18.8%) patients of the isolated DLP, and one (16.7%) case in the combined DLP with CC reconstruction group.

Li et al. [12] also evaluated four types of fixations including the HP and DLP in a total number of 84 patients. Twenty-six cases out of the 84 were treated with HP and 18 patients with anatomical DLP. The HP group reported three cases of osteoarthritis and one patient with shoulder pain. Their overall final outcomes were in favour of the DLP. It was found to have lesser frequency of shoulder pain, reduced rate of complications and more range of motion of the shoulder resulting in early functional recovery in their end results [12]. Li et al. [12] advocated that, given the fact that DLP does not involve the ACJ, therefore, no direct pressure or stress on the rotator cuff and acromion will be applied, hence, there will be no complications such as resorption of the subacromial bone, impingement or injury to the crossing rotator cuff at the subacromial space.

Wang et al. [11] compared 33 patients to 31 patients with HP and DLP, respectively. The follow up results at 12 months postoperatively demonstrated a superiority of DLP in term of lower rate of complications with a significant different on statistical analyses (P<0.05) [11]. In the study, they assessed the preoperative distal clavicular bone block via a three-dimensional computed tomography, this, to guarantee sufficient bone stock for the insertion of adequate distal locking screws as to secure the stability of the fixation [11]. They found that, when the distal fragment has a length of >20mm no loosening occurred post fixation; therefore, they recommend the use of the DLP when the distal fragment is measuring 20mm or more, to ensure sufficient ground for the distal screws [11]. Wang et al. advised the use of DLP when at least four screws could be inserted distally and three proximally [11]. Having the ability to pre-plan the length of the plate and the number of screws, this will help in selecting the shorter plate that could maintain a solid fixation, thus, minimising the length of the scar and the intraoperative soft tissue damage, therefore, a shorter postoperative rehabilitation period and a better functional outcome [11].

Limitations

Limitations to be pointed out in this meta-analysis: First, the relatively small size of the sample may well have influenced the significant differences between the two examined surgical techniques. Second, the mean follow-up (38.7 months for the HP group and 37.03 months for the DLP group) could also be extended to a longer period to have a better prolonged duration of results. And third, low level of evidence of the studies included in the meta-analysis, with only one randomized comparative study in the review. Therefore, the current meta-analysis needs to be revised and the above limitations should be appropriately addressed.

## Conclusions

Contrary to our hypothesis, though HP did show a good radiological outcome (in terms of CCD postoperatively); nonetheless, DLP did demonstrate a better functional result with a lesser rate of complications and the ability to retain the implant which helps the patients in avoiding all the risks that come with a second operation to remove the plate. The initial aim of my study was to help put forward and highlight the beneficial outcomes when resorting to an HP procedure, but practical and scientific proof deemed it rational for me to concur with the DLP method.
